# Supplementary material for: Empirical methods for controlling false positives and estimating confidence in ChIP-Seq peaks
Source: BMC Bioinformatics. 2008 Dec 5;9:523. doi: 10.1186/1471-2105-9-523 (PMC2628906; doi:10.1186/1471-2105-9-523)
Supplement: Additional File 1 — A variety of html documents from the USeq web site detailing the available applications, their best usage, output file type descriptions, command line menus, etc. [file 1471-2105-9-523-S1.zip › USeqUserGuides/JavaDoc.zip!/JavaDoc/edu/utah/seq/methylation454/package-summary.html]

edu.utah.seq.methylation454


|  |  |  |
| --- | --- | --- |
| **Overview** \| **Package** \| Class \| **Use** \| **Tree** \| **Deprecated** \| **Index** \| **Help** | |  |
| **PREV PACKAGE**   **NEXT PACKAGE** | **FRAMES**    **NO FRAMES**     **All Classes** |  |


---

## Package edu.utah.seq.methylation454

Applications for working with 454 data and identifying bisulfite converted bases for methylation studies.

**See:**
  
          **Description**

| **Class Summary** | |
| --- | --- |
| **Amplicon** |  |
| **BaseRead** |  |
| **CpG** |  |
| **DataLine** |  |
| **MethylationParser** |  |
| **Read** | Sequence aligned to a reference. |
| **ReadAlignParser** |  |
| **Reference** |  |
| **Sample** |  |
| **StatMethylationData** |  |

## Package edu.utah.seq.methylation454 Description

Applications for working with 454 data and identifying bisulfite converted bases for methylation studies.

---


|  |  |  |
| --- | --- | --- |
| **Overview** \| **Package** \| Class \| **Use** \| **Tree** \| **Deprecated** \| **Index** \| **Help** | |  |
| **PREV PACKAGE**   **NEXT PACKAGE** | **FRAMES**    **NO FRAMES**     **All Classes** |  |


---
